# Assessment of Substrate Status of Drugs Metabolized by Polymorphic Cytochrome P450 (CYP) 2 Enzymes: An Analysis of a Large-Scale Dataset

**DOI:** 10.3390/biomedicines12010161

**Published:** 2024-01-12

**Authors:** Jakob Sommer, Justyna Wozniak, Judith Schmitt, Jana Koch, Julia C. Stingl, Katja S. Just

**Affiliations:** 1 Institute of Clinical Pharmacology, University Hospital of RWTH Aachen, 52074 Aachen, Germany; jsommer@ukaachen.de (J.S.); jwozniak@ukaachen.de (J.W.); jankoch@ukaachen.de (J.K.); jstingl@ukaachen.de (J.C.S.); 2 Department of Radiology and Biomedical Imaging, Yale University School of Medicine, New Haven, CT 06510, USA

**Keywords:** cytochrome P450 enzyme, CYP2, drug–drug–gene interaction, drug–drug interaction, pharmacogenetics, pharmacogenomics, software solution, database

## Abstract

Background: The analysis of substrates of polymorphic cytochrome P450 (CYP) enzymes is important information to enable drug–drug interactions (DDIs) analysis and the relevance of pharmacogenetics in this context in large datasets. Our aim was to compare different approaches to assess the substrate properties of drugs for certain polymorphic CYP2 enzymes. Methods: A standardized manual method and an automatic method were developed and compared to assess the substrate properties for the metabolism of drugs by CYP2D6, 2C9, and 2C19. The automatic method used a matching approach to three freely available resources. We applied the manual and automatic methods to a large real-world dataset deriving from a prospective multicenter study collecting adverse drug reactions in emergency departments in Germany (ADRED). Results: In total, 23,878 medication entries relating to 895 different drugs were analyzed in the real-world dataset. The manual method was able to assess 12.2% (*n* = 109) of drugs, and the automatic method between 12.1% (*n* = 109) and 88.9% (*n* = 796), depending on the resource used. The CYP substrate classifications demonstrated moderate to almost perfect agreements for CYP2D6 and CYP2C19 (Cohen’s Kappa (κ) 0.48–0.90) and fair to moderate agreements for CYP2C9 (κ 0.20–0.48). Conclusion: A closer look at different classifications between methods revealed that both methods are prone to error in different ways. While the automated method excels in time efficiency, completeness, and actuality, the manual method might be better able to identify CYP2 substrates with clinical relevance.

## 1. Introduction

Pharmacogenomics (PGx) is the study of inherited genetic variants that influence drug efficacy and safety and is believed to be a cornerstone of precision medicine [1]. Thus, besides germline and somatic variants with importance to cancer treatment, germline DNA variations play an important role in the individual drug treatment. Genetic variants can impact individual dose exposures, hence influencing drug safety as well as efficacy [2]. For example, individual clearances of antidepressants and antipsychotics have been shown to vary up to 10-fold based on genetic variants in drug-metabolizing enzymes [3]. Thereby, PGx variants can easily provoke individual effective under- and overdosing of drugs leading to treatment failures and dose-dependent adverse drug reactions (ADR). One important target structure might be phase-I drug metabolizing enzymes of the cytochrome P450 (CYP) system, impacting effective dose exposures. CYP2D6, 2C9, and 2C19 are known to exhibit clinically important PGx polymorphisms [4]. Thus, there are many available guidelines for the clinical implementation of PGx to reduce ADRs and increase efficacy that focus on those CYP2 enzymes [5]. While an association of CYP enzyme variants with the occurrence of ADRs is often assumed [6,7], evidence from clinical studies so far is sparse, but a recent study showed a reduction in the occurrence of ADRs by ~30% with preemptive genotyping, including many CYP variants [8].

Next to PGx variants, CYP enzyme activity can be altered by drug–drug interactions (DDI). Due to drug intake, enzyme inhibition or induction might occur. Therefore, in the case of multi-medication, potential DDIs occur that can likewise impact CYP enzyme activity, as most patients exhibiting ADRs are older and multi-medicated [9]. In the presence of inhibiting DDIs, a subject showing genetically increased CYP enzyme activity can present phenotypically as if the patient had normal or even reduced CYP enzyme activity. This concept of occurring drug–drug–gene interactions is known as phenoconversion [10]. While this phenomenon is a crucial issue to be addressed for the successful implementation of PGx in clinics, evidence of its importance for drug safety and efficacy in large populations is sparse [11].

To generate well-powered evidence on the importance of PGx for ADRs and to deal with DDIs and phenoconversion in large datasets, drugs need to be identified, firstly, as specific CYP enzyme substrates. Clinically, there are software solutions for assessing potential DDIs and some newly developed tools for addressing phenoconversion in patient care [12,13]. However, software solutions for assessing potential DDIs in clinics often produce a problem of overalerting by reporting clinically insignificant DDIs that lead to ignorance of software alerts [14]. Those software solutions can usually be used on individual cases integrated into the electronic health record, as stand-aside software, or as a calculator. Thus, they are usually not capable of analysis of large datasets. On the other hand, several resources offer lists of CYP substrates, inhibitors, and inducers such as the table of substrates, inhibitors, and inducers by the U.S. Food and Drug Administration (FDA) or the Drug Interactions Flockhart Table™ [15,16]. These lists are not conclusive lists for CYP substrates, and it is unclear whether they can be used for the analysis of DDIs and drug–drug–gene interactions in large datasets. Another challenge is the diversity in the biotransformation of drugs: Some drugs are metabolized by a major enzyme and in these drugs, evidence on the PGx variance of CYP enzymes on drug efficacy and safety is most robust [5]. Next to that, there is a large number of drugs that are metabolized via different enzymes, while the extent of CYP involvement is not clear. For some drugs, only in vitro studies are available and the clinical relevance in vivo is unknown. Additionally, some drugs are not metabolized at all.

Hence, to create more evidence on drug safety and efficacy in the case of phenoconversion and multi-medication, approaches are needed to assess the substrate status of drugs. The aim of this study was to compare different approaches for assessing drugs for being CYP2 enzyme substrates in a large-scale dataset.

## 2. Materials and Methods

### 2.1. Data Preparation

We used a large-scale real-world dataset to compare different assessments of the substrate status of drugs. Data from the cohort ‘Adverse Drug Reactions in Emergency Departments’ (ADRED; DRKS-ID: DRKS00008979), a multi-center prospective observational study, were analyzed. Within this dataset, cases of ADRs presenting to four large hospital EDs of tertiary care and academic teaching hospitals were collected between December 2015 and March 2018. Details on study design and enrollment have been published [17]. Patients who were not able to provide written informed consent due to the seriousness of the ADR (e.g., comatose, intubated) were enrolled anonymously and only clinical data were included. Other patients agreed to participate and provided written informed consent. This study was approved by the responsible ethical committee of the University of Bonn (202/15).

Drug use in all cases was documented including the use of over-the-counter drugs (OTC). An anatomical therapeutic chemical (ATC) code was assigned to each drug, that can be used for grouping single substances in drugs [18]. Thus 21,721 medication entries relating to 995 different substance names were documented. Combinational products, such as hydrochlorothiazide/ramipril were split and resulted in two substances. As the ADRED study database was in German, all substances were translated into English. To this end, drugs were matched via their available ATC code to English language databases such as Drugbank or PubChem. Some drugs could not be found by the respective ATC code. This occurred when the documented ATC code was infrequently used or primarily used in Germany, or when the ATC code was only a higher-level ATC code and therefore insufficiently specific to identify a single substance. In case of failure, the search was performed using the substance name of the drug instead of the ATC code. If a substance could not be found based on the name, the script added the letter ‘e’ behind the substance name, since this can often translate substance names from German to English. All other drugs, that could not be translated otherwise, were translated by hand.

### 2.2. Assessments of Substrate Status

We conducted database analyses and analyses of the literature to classify drugs that were used in this real-world dataset as substrates of certain CYP enzymes. We focused our analysis on CYP2 enzymes known for functional and clinically relevant polymorphisms with good evidence levels, namely on CYP2D6, CYP2C9, and CYP2C19 [4,5]. We did so to provide a use case for CYP substrate assessments in which good evidence is available. In addition, there is a certain importance to know the substrate status of drugs where PGx variance impacts drug metabolism and DDIs might occur frequently in clinics.

#### 2.2.1. Manual Assessment of Substrate Status

We set a threshold for the manual assessment of substrate status to include those drugs that were frequently documented in the real-world dataset. Drugs that occurred in at least 3% of all cases were assessed for being a substrate of polymorphic CYP2D6, 2C9, and 2C19. To this end, the drugs were assessed independently by two persons, a clinical pharmacologist, a pharmacist, and/or a medical student in a standardized manner: drug labels, databases such as UpToDate^®^ (www.uptodate.com, accessed on 3 January 2022), PharmGKB (www.pharmgkb.org, accessed on 3 January 2022), and Drugbank (www.drugbank.ca, accessed on 3 January 2022) were checked for information on substrate status [19]. Thereby, focus was laid on resources showing a drug’s substrate status in vivo such as indicated by the drug label or available dosing guidelines on PharmGKB [5]. In cases where it was not clear whether a drug was an enzyme substrate in vivo or in vitro, original articles were checked using PubMed^®^ (www.pubmed.gov, accessed on 3 January 2022). Disagreements between reviewers occurred rarely (~5%). Disagreements in classifications of drugs were discussed between reviewers, a search of the literature was repeated using a dual control principle, and thus a consensus was established.

#### 2.2.2. Automatic Assessment of Substrate Status

An automatic assessment tool was developed in Python 3.10 that matched the English-spelled drugs with different resources for CYP assessment. The automatic assessment tool used three different resources, the publicly available database Drugbank (Version 5.1, exported on 3 January 2022), the U.S. FDA table of examples of clinical substrates (exported 22 September 2023), and the Drug Interactions Flockhart Table^TM^ (exported 22 September 2023) [15,16,19]. Drugbank provides information on the substrate status of numerous medications by collecting study results and is regularly updated. The FDA table is a list of examples intended to guide substrate selection in drug development and interaction studies. The Flockhart Table^TM^ was designed as a reference tool for teaching and research purposes. 

### 2.3. Statistical Analysis

The assessments of drugs between the manual and automatic methods with all three resources were compared. The rate of successful assessments was determined for each method. The unassessed drugs were identified and listed in relation to medication entries, hence, occurrence frequency in the dataset to show limitations in assessments per method and respective resources. Those drugs successfully assessed by both methods were further analyzed. For each drug, it was evaluated whether the manual and automated methods with all three resources reached a consensus in classifying it as a CYP2D6, CYP2C19, or CYP2C9 substrate. The interrater reliability between classifications of both methods was calculated using Cohen’s Kappa and agreements of the two methods for all resources were analyzed [20]. Differences in classifications were compared in relation to their frequency of occurrence in the real-world dataset to show their potential importance in the clinic. The statistical analysis was made in Python 3.10.

## 3. Results

In total, the ADRED-database consisted of 21,721 entries related to 995 different substance names. Those medications were documented in 2939 cases. After splitting combinational products into single drugs, translating substances from German into English, and clearing the database for unspecific drug names, and food supplements, 895 drugs remained for classification, which related to 23,878 medication entries.

### 3.1. Completeness of Assessments

**Figure 1:**
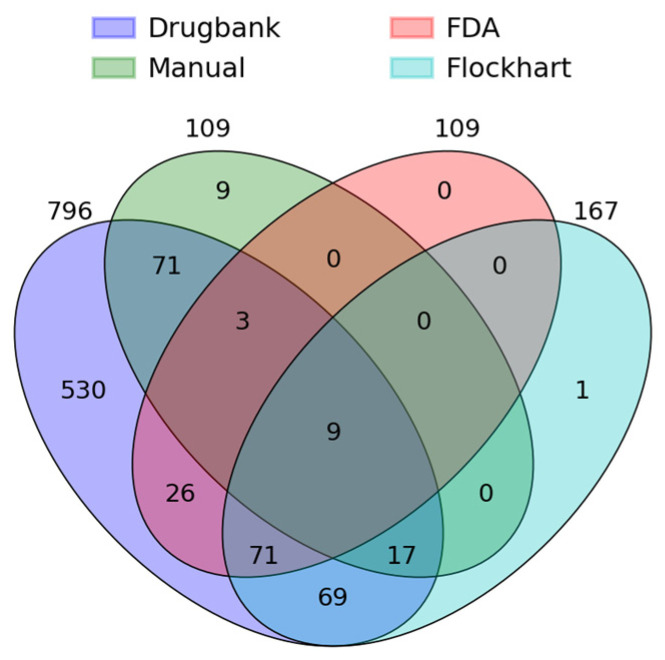
Venn chart showing the different numbers and intersects of drug assessments by a manual and an automatic method using a matching approach to Drugbank, to the FDA Table of Substrates, Inhibitors, and Inducers, and the Flockhart Table^TM^ within a real-world database (ADRED) containing *N* = 895 different drugs.

Out of those *N* = 895 drugs, the manual method assessed 12.2% (*n* = 109), since only drugs taken by ≥3% of ADR cases were analyzed with the manual method. The automatic method was able to classify 88.9% (*n* = 796) of drugs using a matching approach with Drugbank. In the FDA table, *n* = 218 drugs were listed, but not all appeared in the ADRED-database. It was possible to assess 12.1% (*n* = 109) of drugs in the ADRED-database using the automatic method matching with the FDA table. In the Flockhart Table^TM^, *n* = 357 drugs were documented with 18.7% (*n* = 167) of drugs registered in the ADRED-database that could be assessed with this resource. The different proportions of drugs assessed by the two methods with the three resources are shown in Figure 1, and the drugs that remained unassessed are shown in Table 1.

### 3.2. CYP Classification

The manual method classified 9.2% (*n* = 10) of drugs assessed by this method as CYP2D6, 7.3% (*n* = 8) as CYP2C19, and 5.5% (*n* = 6) as CYP2C9 substrates. Using Drugbank, the automatic method resulted in 12.2% (*n* = 97) CYP2D6, 9.8% (*n* = 78) CYP2C19, and 12.3% (*n* = 98) CYP2C9 substrates. With the FDA table, 8.3% (*n* = 9) CYP2D6, 3.7% (*n* = 4) CYP2C19, and again 3.7% (*n* = 4) CYP2C9 substrates were detected in the dataset. Using the Flockhart Table^TM^, 21.6% (*n* = 36) of all drugs assessed with this method could be classified as CYP2D6, 12.6% (*n* = 21) as CYP2C19, and 13.8% (*n* = 23) as CYP2C9 substrates.

The intersections of assessments differed per method and resource used, respectively. In total, *n* = 100 drugs could be classified by both the manual and the automatic assessment method using Drugbank, *n* = 12 drugs using the FDA table, and *n* = 14 drugs using the Flockhart Table^TM^. Frequencies of CYP classification for both methods are shown in Table 2.

Interrater reliability scores differed per method, respective resources, and CYP enzyme. The manual and the automatic method using Drugbank revealed an almost perfect agreement for CYP2D6 (Cohen’s kappa κ = 0.84), a substantial for CYP2C19 (κ = 0.71), and a moderate for CYP2C9 classifications (κ = 0.48). Using the FDA table as a resource for the automatic method showed a moderate agreement for CYP2D6 and CYP2C19 (κ = 0.43), while for CYP2C9 an agreement could not be calculated due to no intersection of both methods. Using the Flockhart Table^TM^ there was an almost perfect agreement with the manual method for CYP2D6 and CYP2C19 (κ = 0.90) and a fair agreement for CYP2C9 (κ = 0.20). Drugs classified differently by the two methods with the three resources are shown in Table 3.

There was no drug that was classified as a specific CYP enzyme by the automatic method with all three resources. Using a matching approach with the FDA table, no drug was classified as a CYP substrate in addition to other resources. One drug, tilidine, was only classified by the manual method as CYP2C19 substrate, but not by the automatic method with none of the resources used. Using the automatic method with a matching approach to Drugbank, *n* = 13 drugs were classified as specific CYP substrates, that were not classified by another resource or method.

## 4. Discussion

This study shows that a manual and an automatic assessment method can be used to classify the involvement of polymorphic CYP2 enzymes in the metabolism of drugs documented in large datasets. However, results can differ largely based on the method used. Differences arise for both methods in the number of possible assessments as well as in classifications of drugs as substrates of polymorphic CYP2 enzymes.

Some method immanent differences occur that need to be respected: The automatic method was able to assess a much higher proportion of entries using a matching approach to Drugbank [19]. The other analyzed resources, the FDA table [15] and the Flockhart Table^TM^ [16], resulted in comparable numbers of assessments as the manual analysis, that focused on the most common drugs. The methods resulted in almost perfect agreements for CYP2D6 and CYP2C19 substrate classifications using the Flockhart Table^TM^ as a resource, but the number of assessed drugs by both methods was low with only 26 out of 895 drugs assessed by both methods. Using the FDA table as a resource for the automatic method resulted in a moderate agreement with the manual method for CYP2D6 and CYP2C19 classifications with even lower numbers of assessments. However, the FDA table is intended to give examples of substrates for DDI studies and therefore is not meant to be conclusive in listing CYP substrates. On the other hand, those drugs listed on the FDA table as CYP substrates have high evidence for substrate attribution with pharmacokinetic studies showing an increase in the area under the curve (AUC) on the administration of an inhibitor [15]. Using a matching approach to Drugbank, the automatic and the manual method had an almost perfect agreement for CYP2D6 and a substantial agreement for CYP2C19 classifications with the highest numbers of assessments by both methods. Regarding CYP2C9 classifications, the manual and the automatic method resulted in only fair agreement using the Flockhart Table^TM^ and moderate using Drugbank. With both resources used by the automatic method, the number of drugs classified as CYP2C9 substrates was higher than with the manual method. A comparison of methods for CYP2C9 classifications using the FDA table was not possible, as those drugs listed in the FDA table as CYP2C9 substrates did not appear in the analyzed dataset. This might be explained by national differences in drug prescriptions, as well as by the nature of a real-world dataset like ADRED. On the other hand, those drugs classified as CYP2C9 substrates by the manual method did not appear in the FDA table.

The divergent interrater reliabilities in CYP substrate classifications represent the conflicting evidence and its interpretation of drug metabolism and its importance in vivo. Concerning the enzyme CYP2D6, both methods resulted in comparable CYP classification with moderate to almost perfect interrater reliability depending on the resource used. The proportion of drugs metabolized via polymorphic CYP2D6 in the ADRED-dataset might be comparable with a published analysis of drugs on the RxList. The latter included 200 drugs frequently used in the United States of America (USA) and resulted in a proportion of 15% [21]. Our dataset consisted of patients admitted for suspected ADRs. Drugs metabolized by polymorphic CYP2 enzymes are frequently expected in ADRs [7,8]. The different proportions per method and resources show the difficulty of classifying a drug clearly as a CYP substrate. Next to that, the differences in proportions may be due to the different datasets used and the number of assessed drugs per method and resource.

As knowledge of the impact of polymorphic CYP enzymes on drug metabolism of certain drugs is a constantly developing field, using an automatic method might be of benefit for a faster day-by-day analysis in case the underlying database is updated regularly. As an example, in previous versions of the manual method, we classified bisoprolol as a CYP2D6 substrate, which resulted in a major difference in the CYP2D6 classification. While the drug metoprolol is unquestionably a CYP2D6 substrate, studies for bisoprolol were in the past not as clear [22]. Bisoprolol seems to be metabolized to a small extent by CYP2D6, but the clinical impact of CYP2D6 PGx variants on the efficacy and safety of bisoprolol is unlikely [23].

For CYP2C19, both methods tested showed moderate to almost perfect agreements. The automatic method using Drugbank was most in line with another study assessing around 10% of drugs as CYP2C19 substrates [21]. This may strengthen the results of the automatic method using Drugbank. In direct comparison to the manual method, the automatic method using Drugbank resulted in more drugs classified as CYP2C19 substrates. This was mainly due to ibuprofen and simvastatin. CYP2C19 is involved in the metabolism of ibuprofen in vitro [24], but so far without clinical relevance [25]. In the case of simvastatin, one review reports CYP2C19 to be involved in the metabolism of some statins including simvastatin [26]. However, no association between CYP2C19 PGx variants and simvastatin effect levels was seen in in vivo studies. So, it can be assumed that CYP2C19 does not affect the metabolism of simvastatin to a clinically relevant extent [27]. Hence, this might be a limitation of the automatic method. With the manual method, more clinically relevant assessments may be possible by focusing on in vivo studies.

In the case of CYP2C9, the resulting proportions diverged largely between the manual and the automatic method with only fair to moderate agreements. Here, ARBs were frequently classified as CYP2C9 substrates in the automatic but not in the manual method. ARBs were shown to be metabolized by CYP2C9 in vitro, but clinical relevance seems to be more complex. In the case of valsartan, for example, it was seen in vitro that the substance is mostly metabolized via CYP2C9, but a minor clinically relevant extent is expected in vivo [28]. However, some studies show clinically relevant differences in the efficacy of certain ARBs (e.g., losartan, irbesartan) based on CYP2C9 PGx variants [29,30]. Hence, different assessments of the clinical relevance of CYP2C9 for the metabolism of ARBs may exist. Consequently, the Flockhart Table^TM^ lists losartan and irbesartan as CYP2C9 substrates, but not other ARBs, as found in Drugbank.

Another example of the divergent classifications and their limitations is that tilidine was only classified by the manual method as a CYP2C19 substrate. Tilidine is metabolized by CYP2C19 and CYP3A4 as shown in vivo [31]. However, this information is not available from the resources used for the automatic method. This might be explained by the very rare use of tilidine and hence its low importance as a drug in the United States and Canada, where the resources used here are edited.

In terms of generalizability, the used dataset is a large-scale, prospective, multicenter study that systematically recorded ADRs in emergency departments. The drugs that were documented in ADRED are comparable to other datasets of ADRs, although international differences might exist [32,33,34]. Within Germany, those drugs documented in the database can typically also be found in similar proportions in everyday hospital care [18]. Hence, even though our methods were used on a dataset of ADR cases, it might be assumed that the external validity is quite high.

In favor of the manual method is the focus on in vivo studies and hence focusing on data that might be clinically relevant in the specific context analyzed. The automatic method may overestimate the role of polymorphic CYP enzymes in drug metabolism for resources such as Drugbank. However, even a minor pathway in drug metabolism could become relevant in the case of multiple DDIs and enzyme saturation in cases of multi-medication. Hence, the decision on what assessment method is best suited should be made based on the research question. On the other hand, the biggest advantage of an automatic method is the time efficiency. Another important aspect might be the objectivity and reliability of the method, updating evidence more often than manageable with a time-consuming manual method. The automatic method is suitable for large datasets that would otherwise be practically impossible to analyze. Due to the extensive time required for manual assessments, for example, it was not feasible to analyze every drug in this real-world dataset. Consequently, we had to establish an arbitrary threshold; here, only drugs with a frequency of use above 3% were included in the analysis. Nevertheless, numerous drugs that were not classified by the manual method due to their low prevalence might have potential clinical significance. The characteristics, benefits, and limitations of different methods are summarized in Table 4.

The performance of the automatic assessment method is highly dependent on the connected database. The Drugbank database, for example, contains a diverse array of studies, but it does not differentiate between major and minor substrates or other clinically relevant properties, such as activity in the central nervous system [19]. This represents a significant limitation and is not aligned with current clinical practice. On the other hand, the FDA table has good evidence for substrates listed but is not conclusive [15]. It might be feasible to use the FDA table for a quick overview analysis aiming to find robust results on CYP substrates. Using the Flockhart Table^TM^ might be a good option to find clinically relevant CYP classifications [16]. For applications in large datasets, the difficulty of how to classify drugs that are not listed remains. However, a pragmatic workaround might be to classify those drugs not specifically listed as not substrates. As of the present, our automatic method could already serve as a screening tool due to its high sensitivity, while eye-catching classifications might be further examined by clinical pharmacologists. The same methods could be used to classify drugs as inhibitors or inducers, but again with the same limitations.

The value of such methods for the analysis of large-scale datasets is high. More evidence on ADRs in the context of multi-medication and PGx variance is needed [35]. To analyze DDIs in real-world data with a focus on PGx variance and DDIs altering drug metabolism, a distinct classification of metabolic pathways is needed that is applicable to large datasets. Relating classifications of the analyzed methods to a large-scale real-world dataset as done here shows that even drugs prescribed frequently in some cases cannot be assessed and classifications can vary based on the method used.

## 5. Conclusions

In conclusion, both automated and manual methods possess distinct advantages and limitations. The selection of the most appropriate method is contingent upon the specific application. It is worth noting that, in terms of clinical significance, the automatic method might tend to overestimate the number of CYP substrates. For a more precise CYP2 substrate assessment, more in vivo studies need to be conducted and integrated into large datasets.

## Figures and Tables

**Table 1:** Top 10 drugs that were not assessed and respective numbers of entries in a real-world dataset per manual and automatic assessment methods with different resources used (*N* = 23,878).

Drug	Manual Assessment Method	Automatic Assessment Method			Entries, *n* (%)
	-	Drugbank	FDA	Flockhart Table^TM^	
pantoprazole	-	-	x	-	1147 (4.8)
acetylsalicylic acid	-	-	x	x	952 (4.0)
torasemide	-	-	x	x	725 (3.0)
ramipril	-	-	x	x	725 (3.0)
levothyroxine	-	-	x	x	619 (2.6)
metamizole	-	-	x	x	567 (2.4)
hydrochlorothiazide	-	-	x	x	454 (1.9)
amlodipine	-	-	x	-	450 (1.9)
potassium	x	-	x	x	419 (1.8)
cholecalciferol	-	-	x	x	388 (1.6)
allopurinol	-	-	-	x	327 (1.4)
phenprocoumon	-	-	-	x	325 (1.4)
magnesium	x	-	-	-	153 (0.6)
formoterol	x	-	-	-	152 (0.6)
tiotropium bromide	-	x	-	-	147 (0.6)
salbutamol	x	-	-	-	122 (0.5)
levodopa	x	-	-	-	120 (0.5)
ipratropium bromide	x	x	-	-	113 (0.5)
oxycodone	x	-	-	-	103 (0.4)
budesonide	x	-	-	-	96 (0.4)
lorazepam	x	-	-	-	85 (0.4)
nitrendipine	x	-	-	-	85 (0.4)
aciclovir	-	x	-	-	53 (0.2)
beclomethasone	-	x	-	-	52 (0.2)
glycerol	-	x	-	-	47 (0.2)
insulin-isophane	-	x	-	-	43 (0.2)
glycopyrronium bromide	-	x	-	-	36 (0.2)
amphotericin b	-	x	-	-	33 (0.1)
ascorbic acid	-	x	-	-	31 (0.1)
potassium iodide	-	x	-	-	29 (0.1)

x: signifies that this drug was under the top 10 drugs not assessed by the respective method. -: signifies that this drug did not occur under the top 10 drugs not assessed by the respective method.

**Table 2:** Comparison of two methods with different resources for classifications of drugs as substrates of polymorphic cytochrome P450 (CYP) enzymes.

	Manual Assessment Method, *n* (%)	Automatic Assessment Method, *n* (%)			Cohen’s Kappa (κ)
	-	Drugbank ^1^	FDA ^2^	Flockhart Table^TM 3^	
CYP2D6	9 (9.0)	12 (12.0)	-	-	0.84
CYP2C19	8 (8.0)	11 (11.0)	-	-	0.71
CYP2C9	6 (6.0)	17 (17.0)	-	-	0.48
CYP2D6	3 (25.0)	-	1 (8.3)	-	0.43
CYP2C19	3 (25.0)	-	1 (8.3)	-	0.43
CYP2C9	0 (0.0)	-	0 (0.0)	-	-
CYP2D6	7 (26.9)	-	-	6 (23.1)	0.90
CYP2C19	7 (26.9)	-	-	6 (23.1)	0.90
CYP2C9	2 (7.7)	-	-	5 (19.2)	0.20

^1^ *n* = 100 drugs were assessed by the manual as well as by the automatic method using Drug Bank. ^2^ *n* = 12 drugs were assessed by the manual as well as by the automatic method using the FDA table. ^3^ *n* = 26 drugs were assessed by the manual as well as by the automatic method using the Flockhart Table^TM^.

**Table 3:** Differences in drug classifications as substrates of polymorphic cytochrome P450 (CYP) enzymes for each automatic assessment method compared to the manual method in relation to medication entries in a real-world dataset (*N* = 23,878).

Enzyme	Drug	Manual Assessment Method	Automatic Assessment Method			Entries, *n* (%)
		-	Drugbank	FDA	Flockhart Table^TM^	
CYP2D6	simvastatin	x	x	-	-	628 (2.6)
	acetaminophen	-	x	NA	-	56 (0.2)
	escitalopram	x	x	-	-	31 (0.1)
	caffeine	-	x	-	-	11 (0.1)
	dapagliflozin	-	x	NA	NA	10 (<0.1)
CYP2C19	simvastatin	-	x	-	-	628 (2.6)
	(dex)ibuprofen	-	x	NA	-	268 (1.1)
	clopidogrel	x	x	-	-	257 (1.1)
	apixaban	x	x	NA	-	218 (0.9)
	naloxone	-	x	NA	NA	137 (0.6)
	tilidine	x	-	NA	NA	110 (0.5)
	escitalopram	x	x	-	-	31 (0.1)
CYP2C9	acetylsalicylic acid	-	x	NA	NA	952 (4.0)
	valsartan	-	x	NA	NA	259 (1.1)
	clopidogrel	-	x	-	x	257 (1.1)
	apixaban	x	x	NA	-	218 (0.9)
	omeprazole	-	x	-	-	121 (0.5)
	carvedilol	-	x	NA	-	97 (0.4)
	ondansetron	-	x	NA	-	87 (0.4)
	losartan	-	x	NA	x	31 (0.1)
	irbesartan	-	x	NA	x	25 (0.1)
	olodaterol	-	x	NA	x	17 (0.1)
	caffeine	-	x	-	-	11 (0.1)
	dapagliflozin	-	x	NA	-	10 (<0.1)

Drugs that were classified by one method as a substrate of polymorphic CYP enzymes, but not by the other are listed comparing automatic with a manual method. x: signifies that the respective method classified the drug as a substrate of the respective enzyme. NA: signifies that the respective method did not assess the drug. -: signifies that the respective method did not classify the drug as a substrate for the respective enzyme.

**Table 4:** Overview of benefits and limitations for the use of different methods to assess and classify drugs as CYP substrates in a real-world dataset.

	Manual Assessment Method	Automatic Assessment Method with Matching Approach to…		
	-	…Drugbank [19]	…FDA [15]	…Flockhart Table^TM^ [16]
Velocity of assessment process	Time consuming	Quick	Quick	Quick
Underlying data	Literature search, standardized approach is recommended for reproducibility of results	In vitro and in vivo data are summarized. CYP substrate classification also based on in vitro data	Data for drugs showing changes in AUC by administration of an inhibitor	Published evidence that a drug is at least in parts metabolized by a CYP enzyme
Dealing with conflicting evidence	Manpower needed for dealing with disagreements. An independent review approach is recommended	No independent reviewers needed, as one clear classification is given	No independent reviewers needed, as one clear classification is given	No independent reviewers needed, as one clear classification is given
Completeness of assessments	Often limited due to time-consuming process needing manpower	Almost complete	Only small number of drugs available	Moderate number of drugs available
Potential clinical relevance of assessments	High with focus on in vivo data	Unclear, as often in vitro data leads to classification of CYP substrates	Very high, as only clear substrates are classified	High with focus on data with evidence in vivo

## Data Availability

The datasets analyzed during the current study are available from the corresponding author on reasonable request. The programming code used for analysis can be found here: https://github.com/Fledermaus12/PathwayQuantification (accessed on 2 January 2024).

## References

[1] Relling M.V., Evans W.E. (2015). Pharmacogenomics in the clinic. Nature.

[2] Hamburg M.A., Collins F.S. (2010). The path to personalized medicine. N. Engl. J. Med..

[3] Stingl J., Brockmöller J., Viviani R. (2013). Genetic variability of drug-metabolizing enzymes: The dual impact on psychiatric therapy and regulation of brain function. Mol. Psychiatry.

[4] Evans W.E., Relling M.V. (1999). Pharmacogenomics: Translating functional genomics into rational therapeutics. Science.

[5] Whirl-Carrillo M., Huddart R., Gong L., Sangkuhl K., Thorn C.F., Whaley R., Klein T.E. (2021). An Evidence-Based Framework for Evaluating Pharmacogenomics Knowledge for Personalized Medicine. Clin. Pharmacol. Ther..

[6] Osanlou O., Pirmohamed M., Daly A.K. (2018). Pharmacogenetics of Adverse Drug Reactions. Adv. Pharmacol..

[7] Meyer U.A. (2000). Pharmacogenetics and adverse drug reactions. Lancet.

[8] Swen J.J., van der Wouden C.H., Manson L.E.N., Abdullah-Koolmees H., Blagec K., Blagus T., Böhringer S., Cambon-Thomsen A., Cecchin E., Cheung K.-C. (2023). A 12-gene pharmacogenetic panel to prevent adverse drug reactions: An open-label, multicentre, controlled, cluster-randomised crossover implementation study. Lancet.

[9] Just K.S., Dormann H., Schurig M., Böhme M., Fracowiak J., Steffens M., Scholl C., Seufferlein T., Gräff I., Schwab M. (2020). Adverse Drug Reactions in the Emergency Department: Is There a Role for Pharmacogenomic Profiles at Risk?—Results from the ADRED Study. J. Clin. Med..

[10] Shah R.R., Smith R.L. (2015). Addressing phenoconversion: The Achilles’ heel of personalized medicine. Br. J. Clin. Pharmacol..

[11] Klomp S.D., Manson M.L., Guchelaar H.J., Swen J.J. (2020). Phenoconversion of Cytochrome P450 Metabolism: A Systematic Review. J. Clin. Med..

[12] Cicali E.J., Elchynski A.L., Cook K.J., Houder J.T., Thomas C.D., Smith D.M., Elsey A., Johnson J.A., Cavallari L.H., Wiisanen K. (2021). How to Integrate CYP2D6 Phenoconversion Into Clinical Pharmacogenetics: A Tutorial. Clin. Pharmacol. Ther..

[13] Kheshti R., Aalipour M., Namazi S. (2016). A comparison of five common drug-drug interaction software programs regarding accuracy and comprehensiveness. J. Res. Pharm. Pract..

[14] Kuperman G.J., Bobb A., Payne T.H., Avery A.J., Gandhi T.K., Burns G., Classen D.C., Bates D.W. (2007). Medication-related clinical decision support in computerized provider order entry systems: A review. J. Am. Med. Inform. Assoc..

[15] FDA Drug Development and Drug Interactions: Table of Substrates, Inhibitors, and Inducers. https://www.fda.gov/drugs/drug-interactions-labeling/drug-development-and-drug-interactions-table-substrates-inhibitors-and-inducers.

[16] Flockhart D.A. Drug Interactions Flockhart Table™. https://drug-interactions.medicine.iu.edu/MainTable.aspx.

[17] Schurig A.M., Bohme M., Just K.S., Scholl C., Dormann H., Plank-Kiegele B., Seufferlein T., Graff I., Schwab M., Stingl J.C. (2018). Adverse Drug Reactions (ADR) and Emergencies. Dtsch. Arztebl. Int..

[18] Just K.S., Dormann H., Böhme M., Schurig M., Schneider K.L., Steffens M., Dunow S., Plank-Kiegele B., Ettrich K., Seufferlein T. (2020). Personalising drug safety—Results from the multi-centre prospective observational study on Adverse Drug Reactions in Emergency Departments (ADRED). Eur. J. Clin. Pharmacol..

[19] Wishart D.S., Feunang Y.D., Guo A.C., Lo E.J., Marcu A., Grant J.R., Sajed T., Johnson D., Li C., Sayeeda Z. (2018). DrugBank 5.0: A major update to the DrugBank database for 2018. Nucleic Acids Res..

[20] Landis J.R., Koch G.G. (1977). The measurement of observer agreement for categorical data. Biometrics.

[21] Zanger U.M., Turpeinen M., Klein K., Schwab M. (2008). Functional pharmacogenetics/genomics of human cytochromes P450 involved in drug biotransformation. Anal. Bioanal. Chem..

[22] Castaño-Amores C., Díaz-Villamarín X., Pérez-Gutiérrez A.M., Antúnez-Rodríguez A., Pozo-Agundo A., Moreno-Escobar E., Sánchez-Ramos J.G., Martínez-González L.J., Dávila-Fajardo C.L. (2021). Pharmacogenetic polymorphisms affecting bisoprolol response. Biomed. Pharmacother..

[23] KNMP CYP2D6: Bisoprolol. https://www.g-standaard.nl/risicoanalyse/B0002457.PDF.

[24] Chang S.Y., Li W., Traeger S.C., Wang B., Cui D., Zhang H., Wen B., Rodrigues A.D. (2008). Confirmation that cytochrome P450 2C8 (CYP2C8) plays a minor role in (S)-(+)- and (R)-(−)-ibuprofen hydroxylation in vitro. Drug Metab. Dispos..

[25] Saiz-Rodríguez M., Valdez-Acosta S., Borobia A.M., Burgueño M., Gálvez-Múgica M., Acero J., Cabaleiro T., Muñoz-Guerra M.F., Puerro M., Llanos L. (2021). Influence of Genetic Polymorphisms on the Response to Tramadol, Ibuprofen, and the Combination in Patients with Moderate to Severe Pain after Dental Surgery. Clin. Ther..

[26] Kitzmiller J.P., Mikulik E.B., Dauki A.M., Murkherjee C., Luzum J.A. (2016). Pharmacogenomics of statins: Understanding susceptibility to adverse effects. Pharmacogenom. Pers. Med..

[27] Choi H.Y., Bae K.S., Cho S.H., Ghim J.L., Choe S., Jung J.A., Jin S.J., Kim H.S., Lim H.S. (2015). Impact of CYP2D6, CYP3A5, CYP2C19, CYP2A6, SLCO1B1, ABCB1, and ABCG2 gene polymorphisms on the pharmacokinetics of simvastatin and simvastatin acid. Pharmacogenet. Genom..

[28] Nakashima A., Kawashita H., Masuda N., Saxer C., Niina M., Nagae Y., Iwasaki K. (2005). Identification of cytochrome P450 forms involved in the 4-hydroxylation of valsartan, a potent and specific angiotensin II receptor antagonist, in human liver microsomes. Xenobiotica.

[29] Hallberg P., Karlsson J., Kurland L., Lind L., Kahan T., Malmqvist K., Ohman K.P., Nyström F., Melhus H. (2002). The CYP2C9 genotype predicts the blood pressure response to irbesartan: Results from the Swedish Irbesartan Left Ventricular Hypertrophy Investigation vs Atenolol (SILVHIA) trial. J. Hypertens..

[30] Joy M.S., Dornbrook-Lavender K., Blaisdell J., Hilliard T., Boyette T., Hu Y., Hogan S.L., Candiani C., Falk R.J., Goldstein J.A. (2009). CYP2C9 genotype and pharmacodynamic responses to losartan in patients with primary and secondary kidney diseases. Eur. J. Clin. Pharmacol..

[31] Grün B., Krautter S., Riedel K.D., Mikus G. (2009). Inhibition of the active principle of the weak opioid tilidine by the triazole antifungal voriconazole. Br. J. Clin. Pharmacol..

[32] Budnitz D.S., Shehab N., Kegler S.R., Richards C.L. (2007). Medication use leading to emergency department visits for adverse drug events in older adults. Ann. Intern. Med..

[33] Shehab N., Lovegrove M.C., Geller A.I., Rose K.O., Weidle N.J., Budnitz D.S. (2016). US emergency department visits for outpatient adverse drug events, 2013–2014. JAMA.

[34] Pirmohamed M., James S., Meakin S., Green C., Scott A.K., Walley T.J., Farrar K., Park B.K., Breckenridge A.M. (2004). Adverse drug reactions as cause of admission to hospital: Prospective analysis of 18,820 patients. BMJ.

[35] Just K.S., Dormann H., Freitag M., Schurig M., Böhme M., Steffens M., Scholl C., Seufferlein T., Graeff I., Schwab M. (2021). CYP2D6 in the Brain: Potential Impact on Adverse Drug Reactions in the Central Nervous System-Results From the ADRED Study. Front. Pharmacol..

